# Supplementary material for: Graph Topology Reframes the Coherence of Cell-State Manifold Inference under Heterogeneous Single-Cell Observations
Source: Comput Struct Biotechnol J. 2026 Jun 3;35(1):0087. doi: 10.34133/csbj.0087 (PMC13230998; doi:10.34133/csbj.0087)

Supplementary Figure 1 (Not interactive)

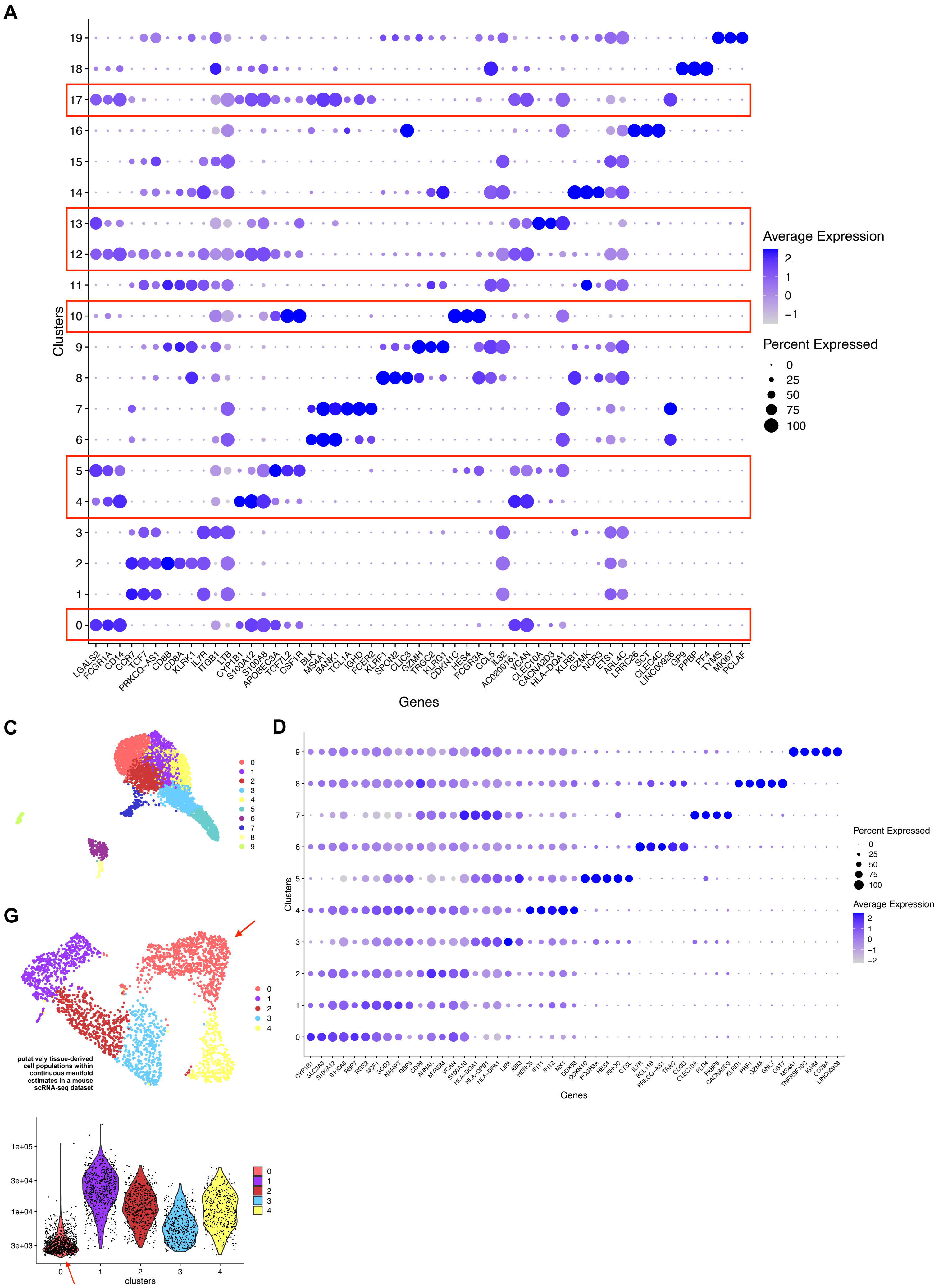

Supplementary Figure 2 (Not interactive)

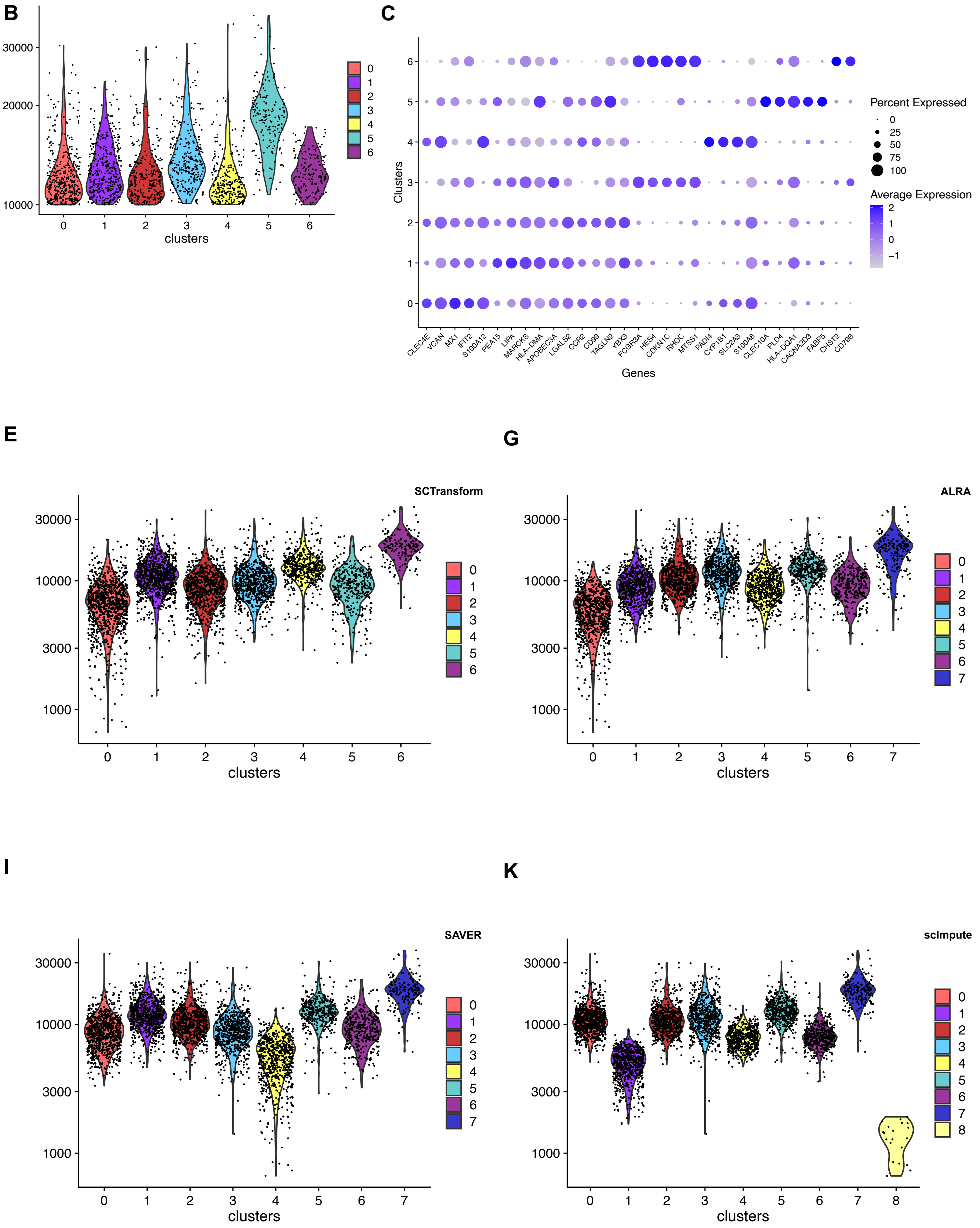

Supplementary Figure 3 (Not interactive)

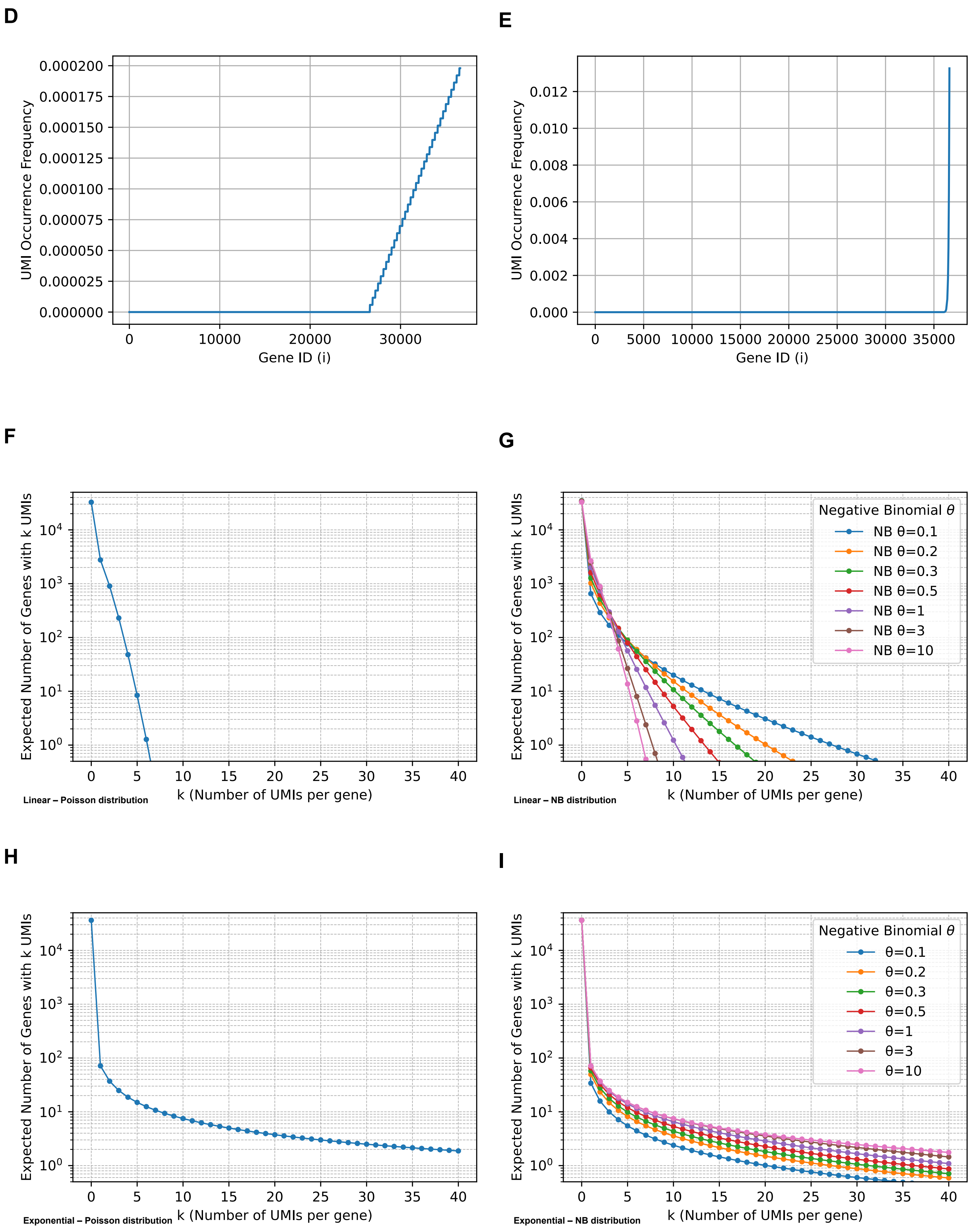

Supplement: Supplementary 1 — Figs. S1 to S4 [file csbj.0087.f1.zip › Supplementary Figures/Supplementary Figures (not interactive).pdf]
