# Supplementary material for: Graph Topology Reframes the Coherence of Cell-State Manifold Inference under Heterogeneous Single-Cell Observations
Source: Comput Struct Biotechnol J. 2026 Jun 3;35(1):0087. doi: 10.34133/csbj.0087 (PMC13230998; doi:10.34133/csbj.0087)
Supplement: Supplementary 1 — Figs. S1 to S4 [file csbj.0087.f1.zip › Supplementary Figure Legends V3.docx]

**Supplementary Figure Legends**

**Supplementary Figure 1. Empirical demonstration of the mechanism by which shallow observation distorts data manifold interpretations.**

**A** Dot plot showing genes highly expressed in each cluster, as identified by differential expression analysis across the entire population of viable PBMCs. Dot color indicates normalized expression levels, and dot size represents the proportion of cells expressing each gene.

**B** Interactive HTML-based visualization of dot plots visualizing the three-dimensional distribution of monocyte *raw* populations (clusters 0, 4, 5, 10, 12, 13, and 17), with UMAP coordinates on the x–y plane and total UMI counts on the z-axis.

**C** From the entire population of viable PBMCs, we extracted the monocyte *raw* populations, and applied the workflow (#). The UMAP plot is shown with the resulting cluster annotations.

**D** Dot plot showing genes highly expressed in each cluster, as identified by differential expression analysis across the monocyte *raw* populations reanalyzed by workflow (#). Dot color indicates normalized expression levels, and dot size represents the proportion of cells expressing each gene.

**E** Interactive HTML-based visualization of dot plots visualizing the three-dimensional distribution of doublet-removed, putative monocyte population $M$, with UMAP coordinates on the x–y plane and total UMI counts on the z-axis.

**F** Interactive HTML-based visualization of dot plots visualizing the three-dimensional distribution of putatively tissue-derived (low unmapped read ratio; #UnmappedRead / #TotalRead$<1.0$) mouse cell population within continuous manifold estimates (assigned to an identical cluster when clustering whole mouse cell population; $\%$neighbor cells$=2.0\%$, resolution$=0.05$, using $10$ principal components), with UMAP coordinates on the x–y plane and total UMI counts on the z-axis.

**G** The UMAP plot and violin plot of total UMI counts per cell in each cluster of the putatively tissue-derived mouse cell population within continuous manifold estimates. An aggregate of shallowly observed cells (cluster 0) is indicated by a red arrow.

**Supplementary Figure 2. Graph-based manifold reconstructions using high-information cells alone generate a low-dimensional structure representing cell state transition with biological constraints.**

**A** Interactive HTML-based visualization of dot plots visualizing the three-dimensional distribution of high-information cell-restricted monocyte population $M'$, with UMAP coordinates on the x–y plane and total UMI counts on the z-axis.

**B** Violin plot of total UMI counts per cell in each cluster of $M'$.

**C** Dot plot showing genes highly expressed in each cluster, as identified by differential expression analysis across $M'$. Dot color indicates normalized expression levels, and dot size represents the proportion of cells expressing each gene.

**D** Interactive HTML-based visualization of dot plots visualizing the three-dimensional distribution of SCTransform-normalized $M$, with UMAP coordinates on the x–y plane and total UMI counts on the z-axis.

**E** Violin plot of total UMI counts per cell in each cluster of SCTransform-normalized $M$.

**F** Interactive HTML-based visualization of dot plots visualizing the three-dimensional distribution of ALRA-imputed $M$, with UMAP coordinates on the x–y plane and total UMI counts on the z-axis.

**G** Violin plot of total UMI counts per cell in each cluster of ALRA-imputed $M$.

**H** Interactive HTML-based visualization of dot plots visualizing the three-dimensional distribution of SAVER-imputed $M$, with UMAP coordinates on the x–y plane and total UMI counts on the z-axis.

**I** Violin plot of total UMI counts per cell in each cluster of SAVER-imputed $M$.

**J** Interactive HTML-based visualization of dot plots visualizing the three-dimensional distribution of scImpute-imputed $M$, with UMAP coordinates on the x–y plane and total UMI counts on the z-axis.

**K** Violin plot of total UMI counts per cell in each cluster of scImpute-imputed $M$.

**Supplementary Figure 3. Detailed and interactive visualization of computational simulations for how heterogeneous single-cell observation influences interpretations.**

**A** Interactive HTML-based visualization of the number of genes $N(k)$with exactly $k$observed UMIs in the empirical peripheral blood mononuclear cell scRNA-seq data deposited by 10x Genomics (Human; in vivo; 36,601 genes; 11,996 cells). We stratified cells by their total UMI counts into bins of width 1,000 (cells with total UMI counts from $1,000$ to $11,999$ are shown) and visualized $\bar{N}(k)$, the mean of $N(k)$, for each bin.

**B** Interactive HTML-based visualization of the number of genes $N(k)$with exactly $k$observed UMIs in the empirical lung progenitor organoid scRNA-seq data deposited by Y. Miao *et al* (Human; in vitro; $36,601$ genes; $14,100$ cells). We stratified cells by their total UMI counts into bins of width $1,000$ (cells with total UMI counts from $1,000$ to $11,999$ are shown) and visualized $\bar{N}(k)$, the mean of $N(k)$, for each bin.

**C** Interactive HTML-based visualization of the number of genes $N(k)$with exactly $k$observed UMIs in the empirical benchmark scRNA-seq data deposited by E. Mereu *et al* (Mouse; complex sample; $34,900$ genes; $4,996$ cells). We stratified cells by their total UMI counts into bins of width $1,000$ (cells with total UMI counts from $1,000$ to $11,999$ are shown) and visualized $\bar{N}(k)$, the mean of $N(k)$, for each bin.

**D** A linear distribution model for the composition ratio $p$ of gene-derived products in the cDNA pool. The number of genes is set to $36,601$, corresponding to a typical gene panel. For each gene, we model the molecule count as an integer so that the total number of molecules is on the order of ${10}^{5}$, and we use the values normalized by the total molecule count as $p$. Genes are indexed such that ​$p_{1}\leq p_{2}\leq\cdots\leq p_{36,601}$. It is generally considered that approximately $10,000$ genes are expressed in a single cell; in this model, gene expression is allowed only for $i\geq26,601$. For $26,602\leq i\leq36601$, $p_{i}$ increases linearly.

**E** An exponential distribution model for the composition ratio $p$ of gene-derived products in the cDNA pool. The number of genes is set to $36,601$, corresponding to a typical gene panel. For each gene, we model the molecule count as an integer so that the total number of molecules is on the order of ${10}^{5}$, and we use the values normalized by the total molecule count as $p$. Genes are indexed such that ​$p_{1}\leq p_{2}\leq\cdots\leq p_{36,601}$. It is generally considered that approximately $10,000$ genes are expressed in a single cell; in this model, gene expression is allowed only for $i\geq26,601$. For $i\geq36,000$, $p_{i}$ increases exponentially.

**F** Line plot showing the expected number of genes $N(k)$with exactly $k$observed UMIs under a Poisson sampling model from the linear distribution model for $p_{i}$. For each gene, all UMIs are assumed to be sampled with equal probability, and a different UMI is selected at each draw.

**G** Line plots showing the expected $N(k)$ under a negative binomial sampling model from the linear distribution model for $p_{i}$. Expectations are computed for the overdispersion parameter $\theta=0.1$, $0.3$, $0.5$, $1$, $3$, and $10$.

**H** Line plot showing the expected number of genes $N(k)$with exactly $k$observed UMIs under a Poisson sampling model from the exponential distribution model for $p_{i}$. For each gene, all UMIs are assumed to be sampled with equal probability, and a different UMI is selected at each draw.

**I** Line plots showing the expected $N(k)$ under a negative binomial sampling model from the exponential distribution model for $p_{i}$. Expectations are computed for the overdispersion parameter $\theta=0.1$, $0.3$, $0.5$, $1$, $3$, and $10$.

**J** Interactive HTML-based visualization of dot plots visualizing the three-dimensional distribution of simulated cell population $X$, with UMAP coordinates on the x–y plane and total UMI counts on the z-axis.

**K** Interactive HTML-based visualization of dot plots visualizing the three-dimensional distribution of simulated cell population $Y$, with UMAP coordinates on the x–y plane and total UMI counts on the z-axis.

**L** Interactive HTML-based visualization of dot plots visualizing the three-dimensional distribution of simulated cell population $Z$, with UMAP coordinates on the x–y plane and total UMI counts on the z-axis.

**Supplementary Figure 4. Biologically significant differences in basal transcriptomic activities are ignored by too simple thresholding by observation depth.**

Interactive HTML-based visualization of dot plots visualizing the three-dimensional distribution of the entire population of viable PBMC including various cell types such as not only monocytes but also lymphocytes and other blood cells, with UMAP coordinates on the x–y plane and total UMI counts on the z-axis.
