# Supplementary material for: Graph Topology Reframes the Coherence of Cell-State Manifold Inference under Heterogeneous Single-Cell Observations
Source: Comput Struct Biotechnol J. 2026 Jun 3;35(1):0087. doi: 10.34133/csbj.0087 (PMC13230998; doi:10.34133/csbj.0087)

Supplementary Figure 1 (Part 1, not interactive)

*T. Tamura, *Y. Yamane, *et al.*

A

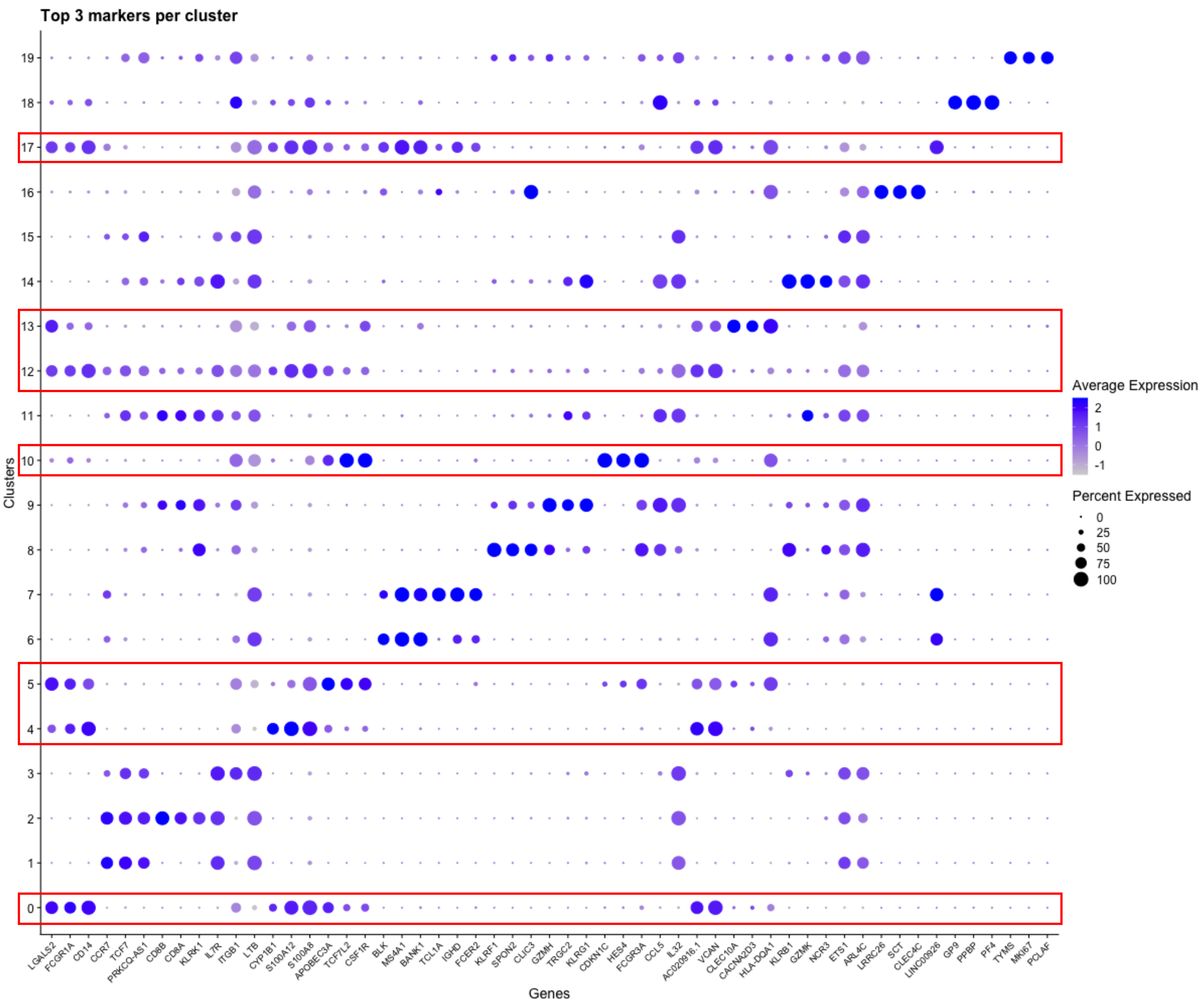

Supplementary Figure 1 (Part 2, not interactive)

*T. Tamura, *Y. Yamane, *et al.*

C

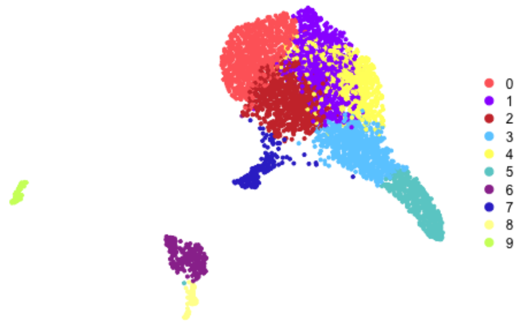

D

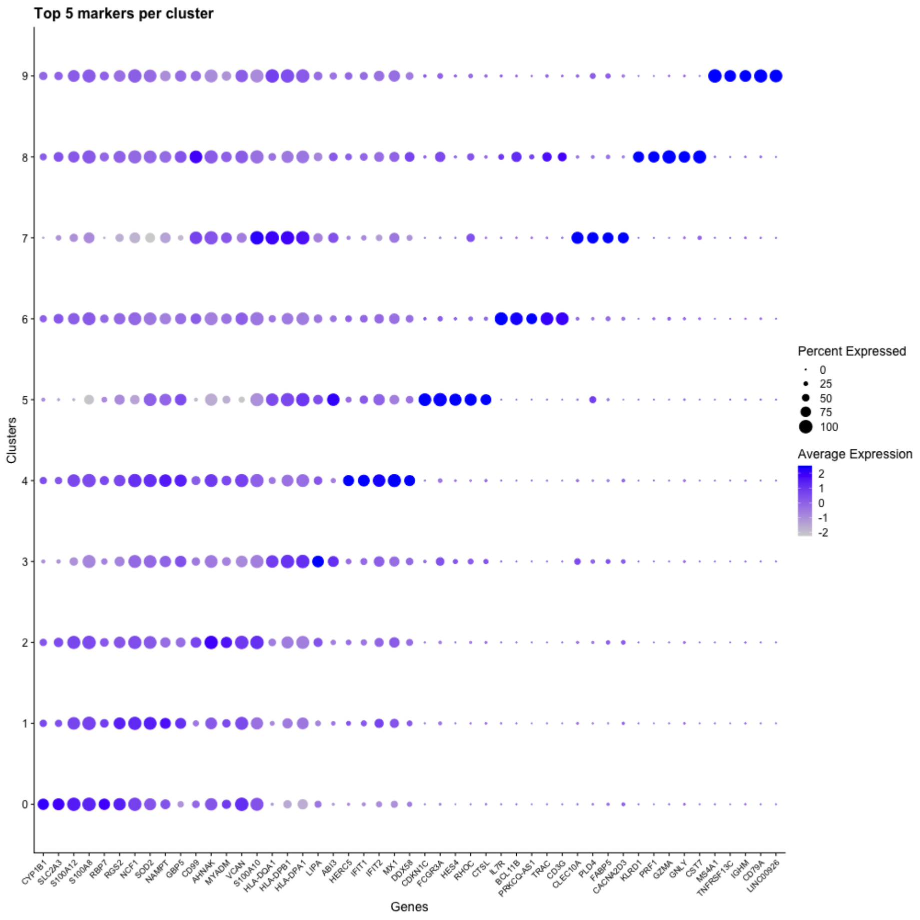

G putatively tissue-derived cell populations within continuous manifold estimates in a mouse scRNA-seq dataset

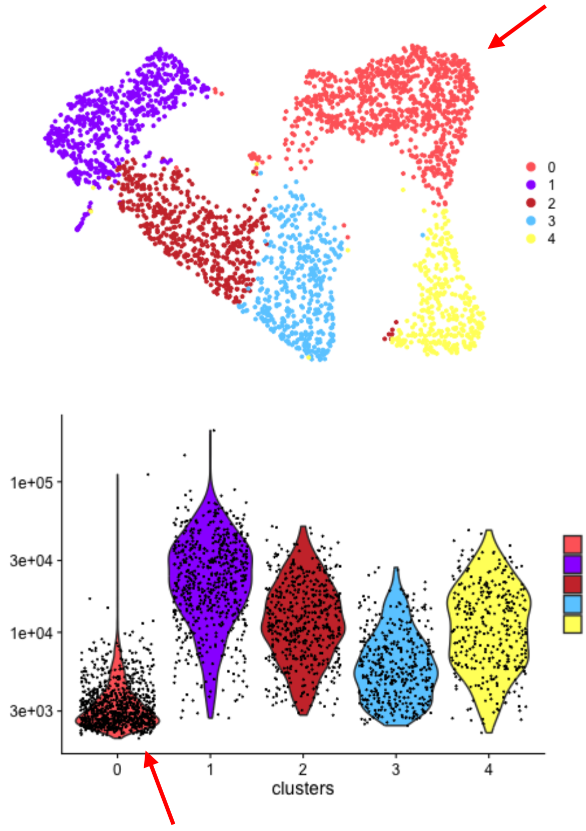

Supplementary Figure 2 (not interactive)

*T. Tamura, *Y. Yamane, *et al.*

B

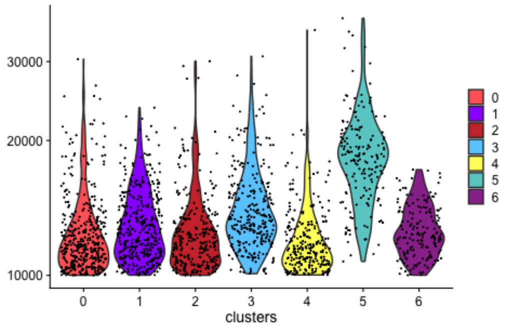

C

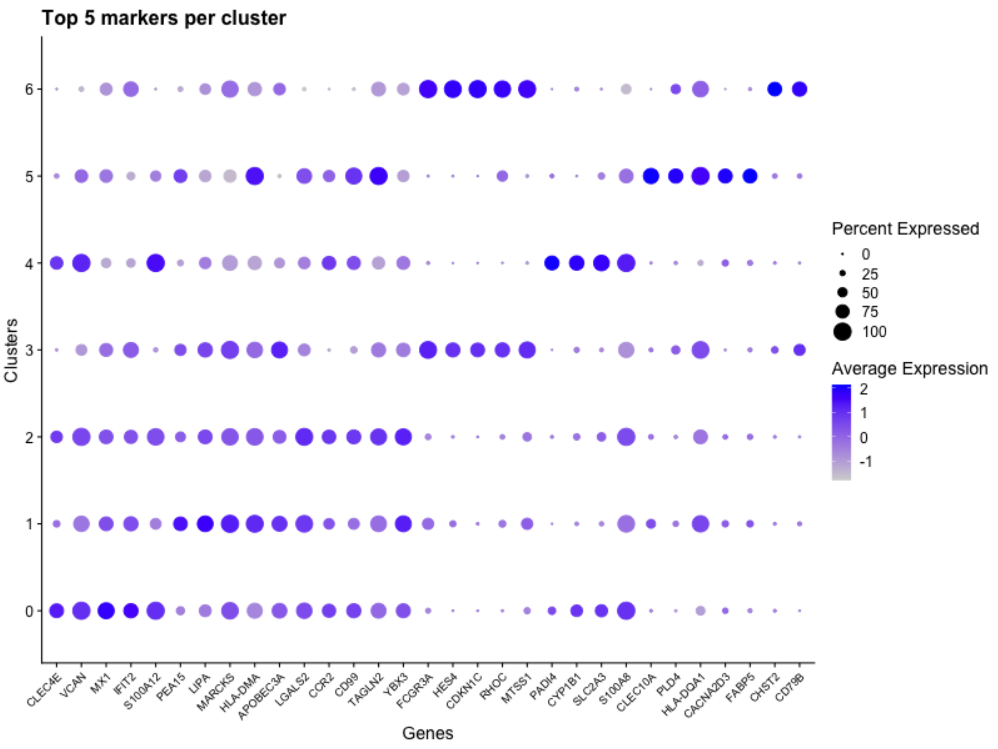

E

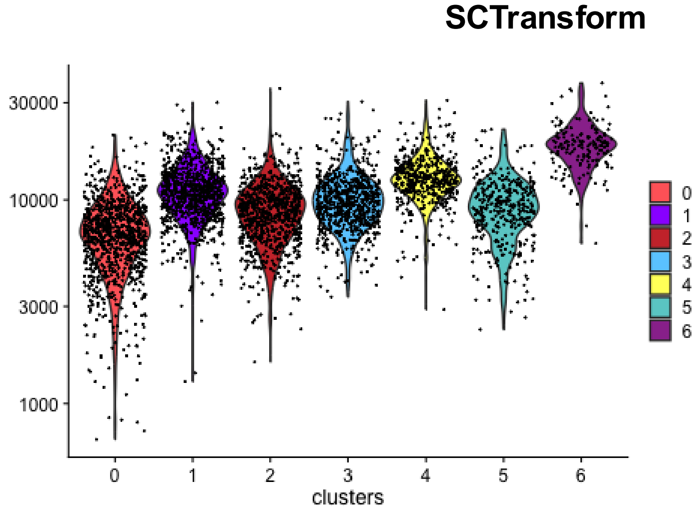

G

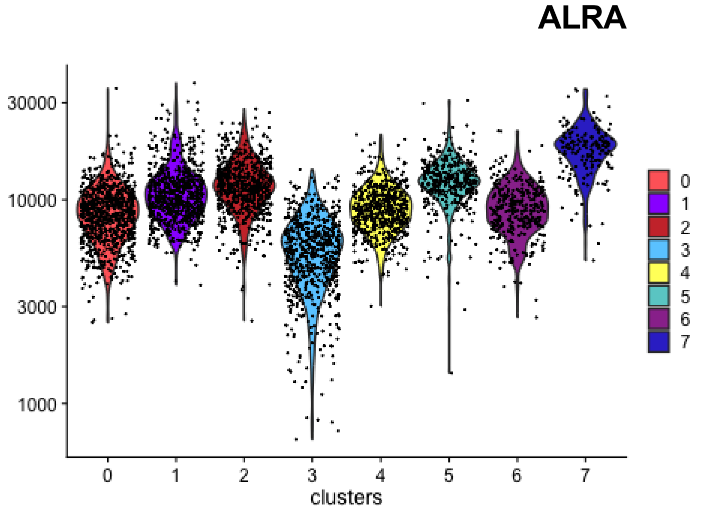

I

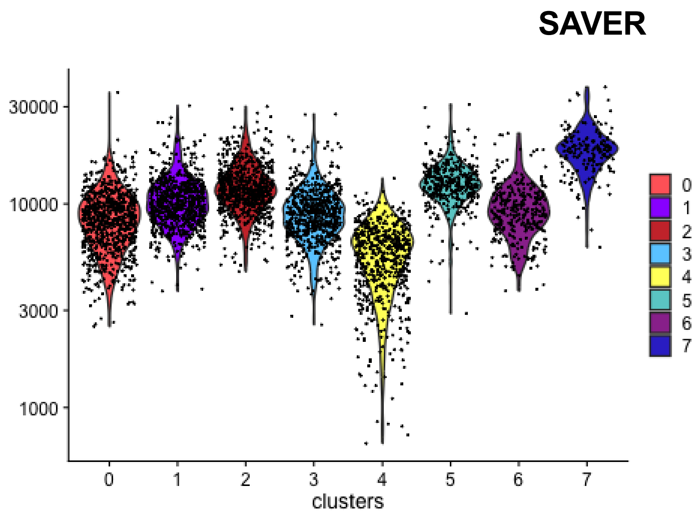

K

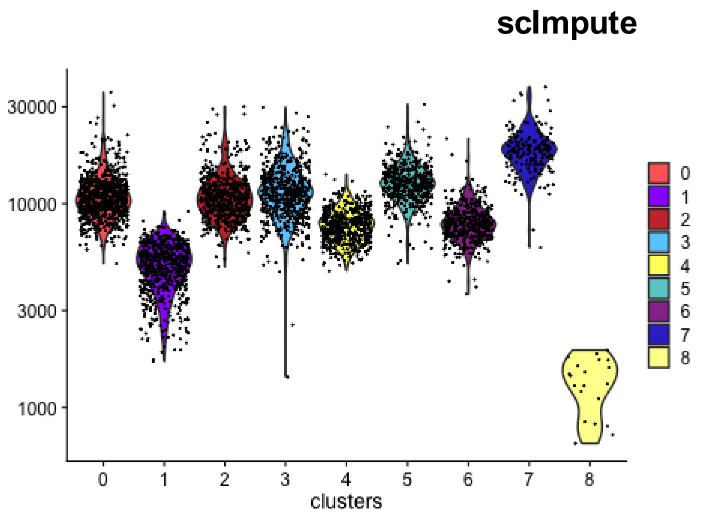

Supplementary Figure 3 (not interactive)

*T. Tamura, *Y. Yamane, *et al.*

D

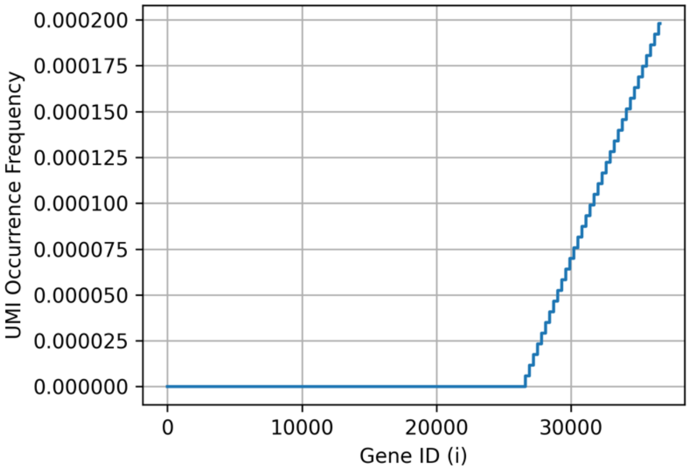

E

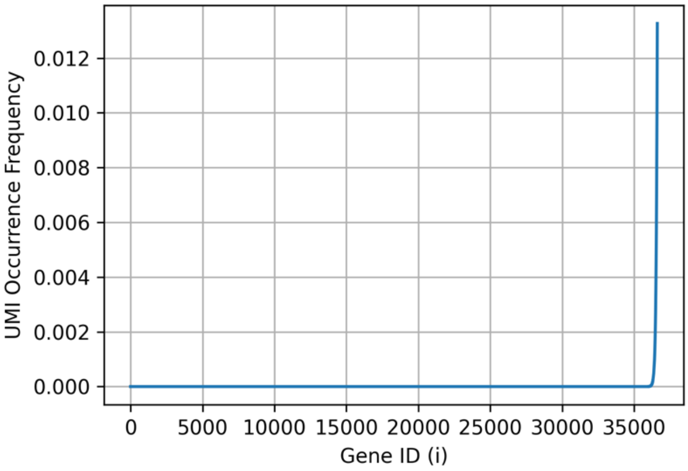

F

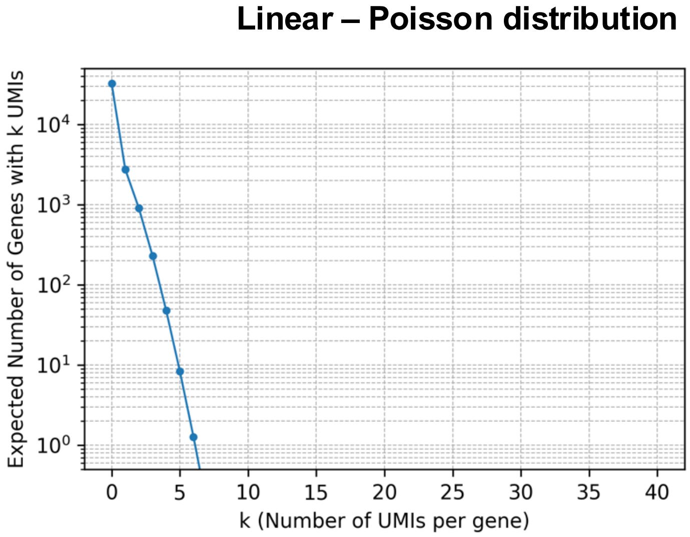

G

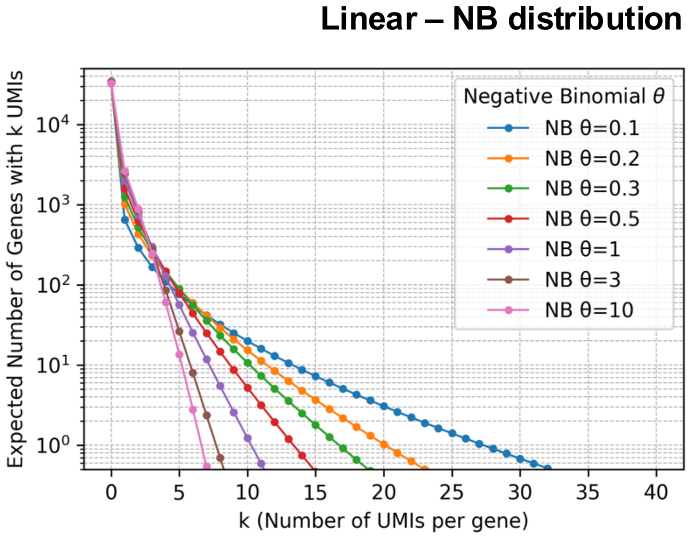

H

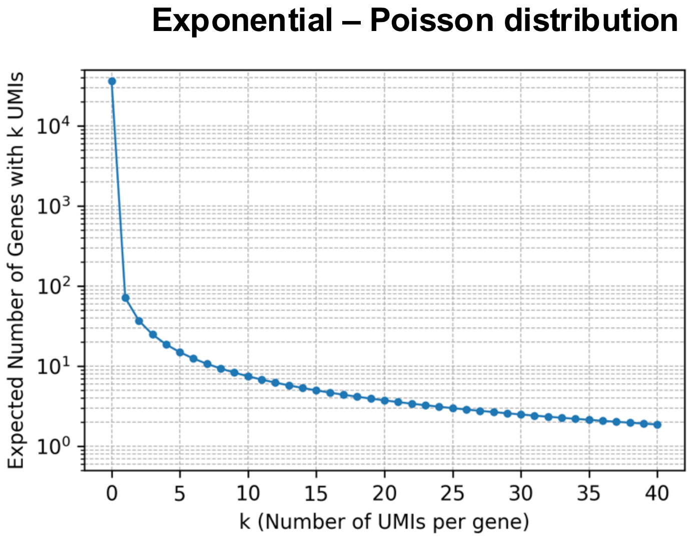

I

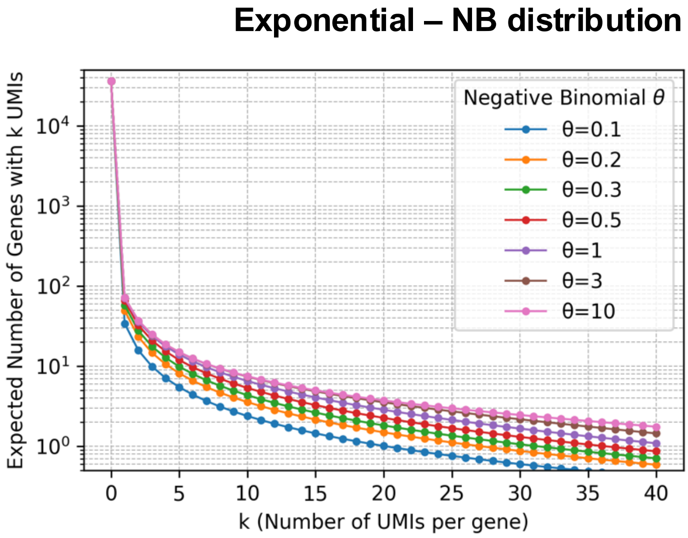

Supplement: Supplementary 1 — Figs. S1 to S4 [file csbj.0087.f1.zip › Supplementary Figures_not interactive.pdf]
