# Supplementary figures and images for: Graph Topology Reframes the Coherence of Cell-State Manifold Inference under Heterogeneous Single-Cell Observations
Source: Comput Struct Biotechnol J. 2026 Jun 3;35(1):0087. doi: 10.34133/csbj.0087 (PMC13230998; doi:10.34133/csbj.0087)

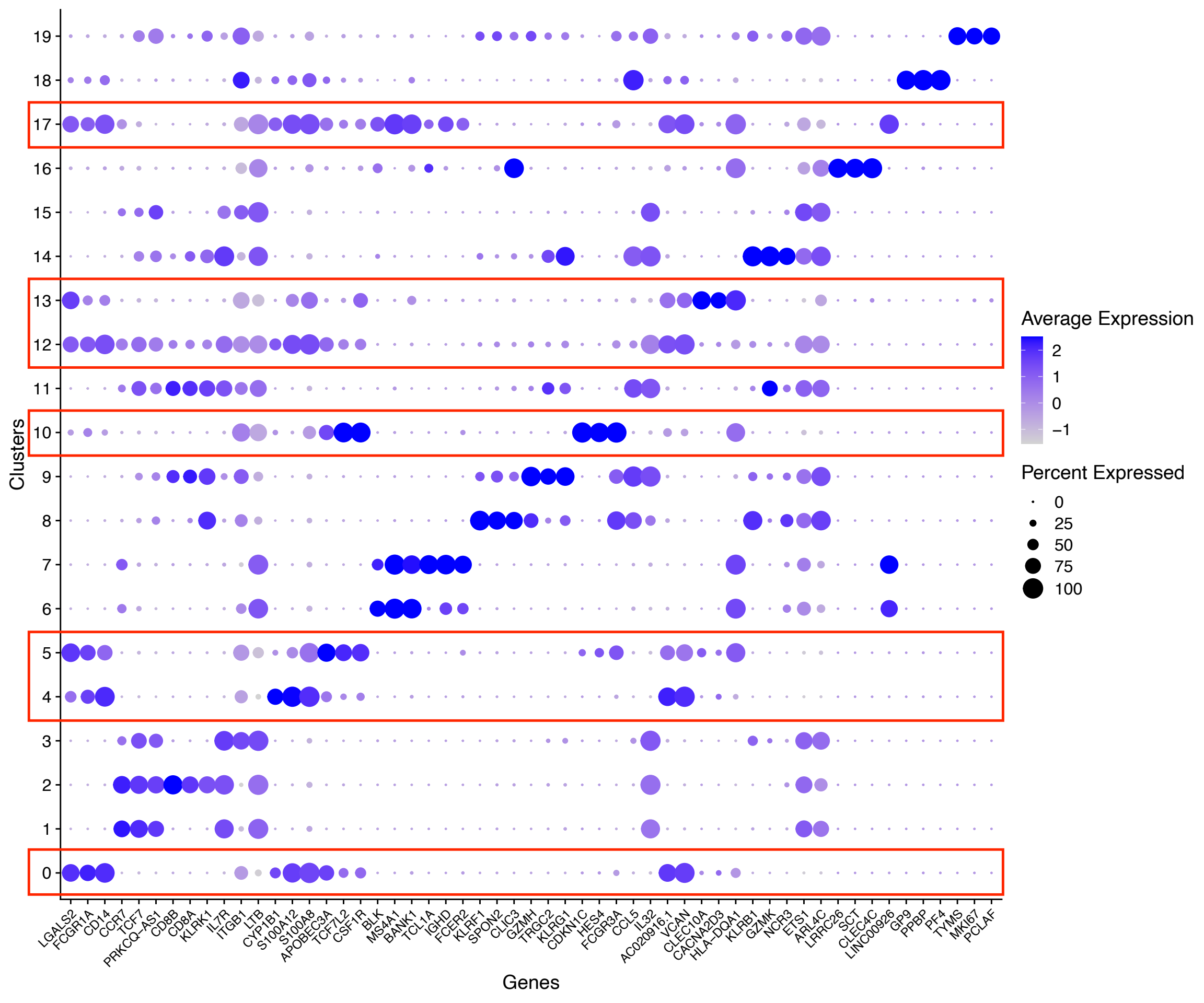

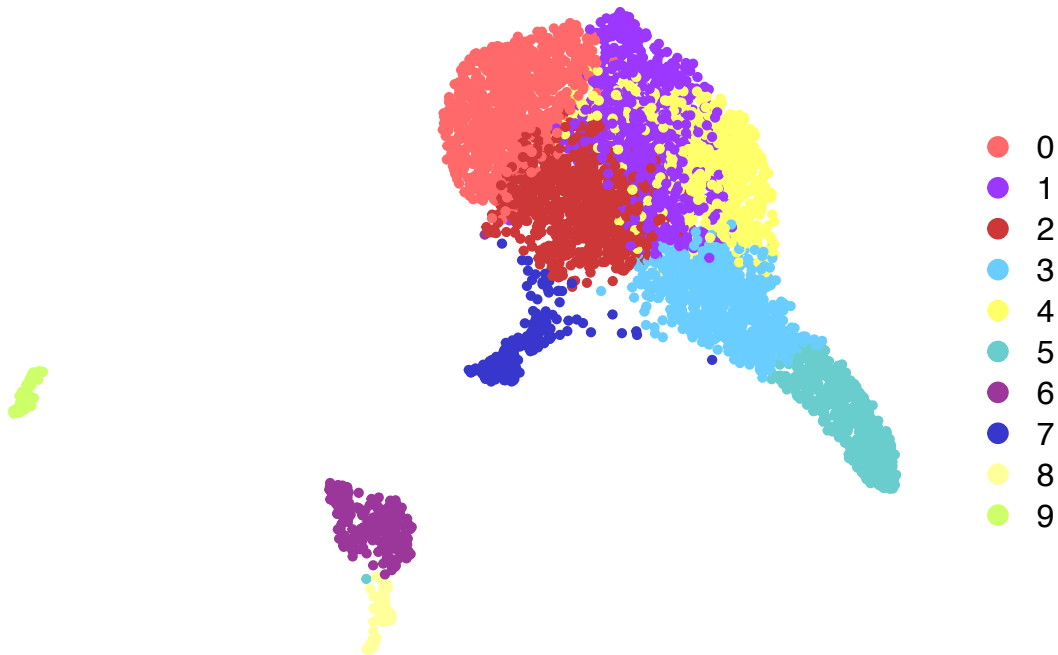

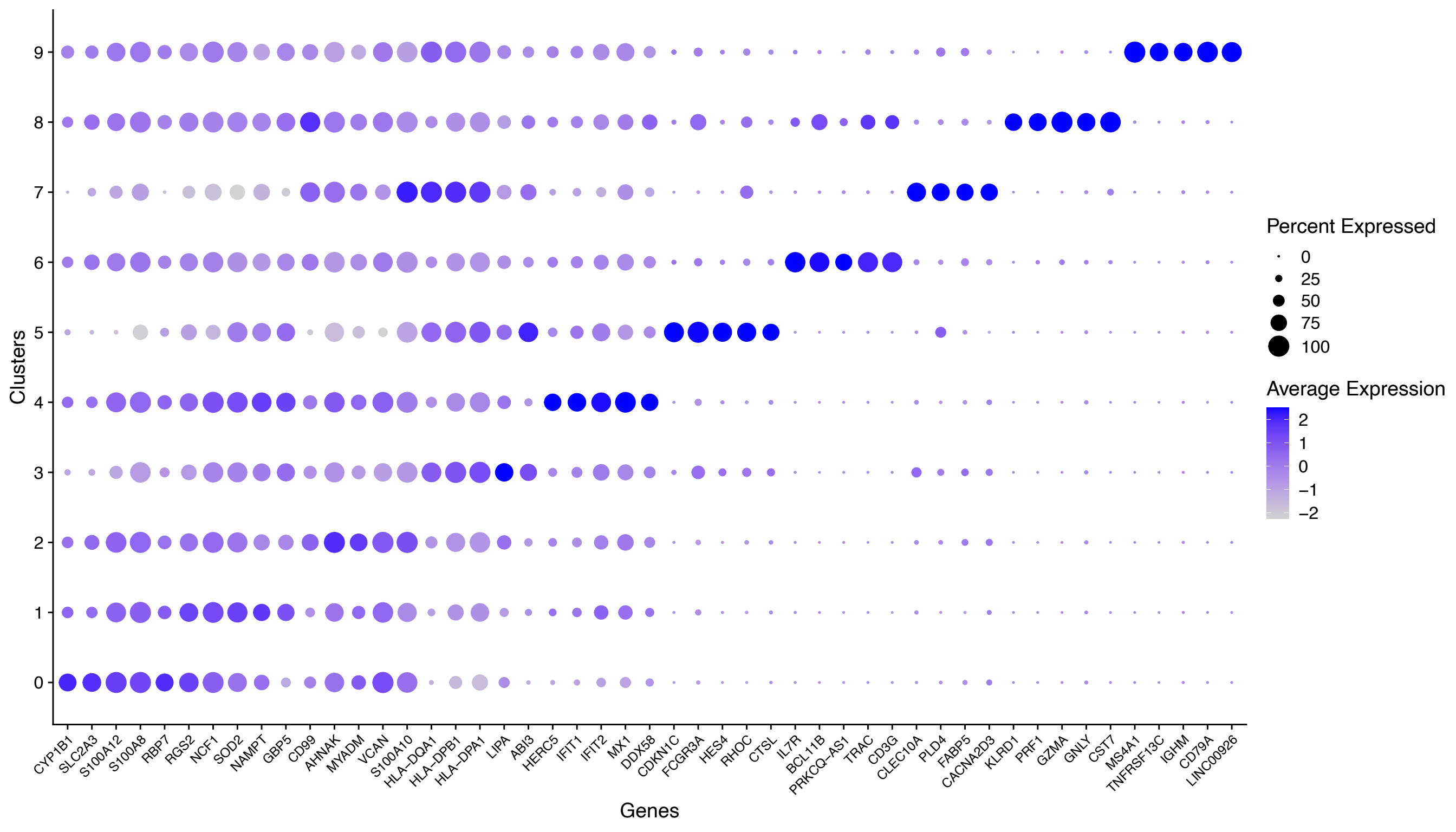

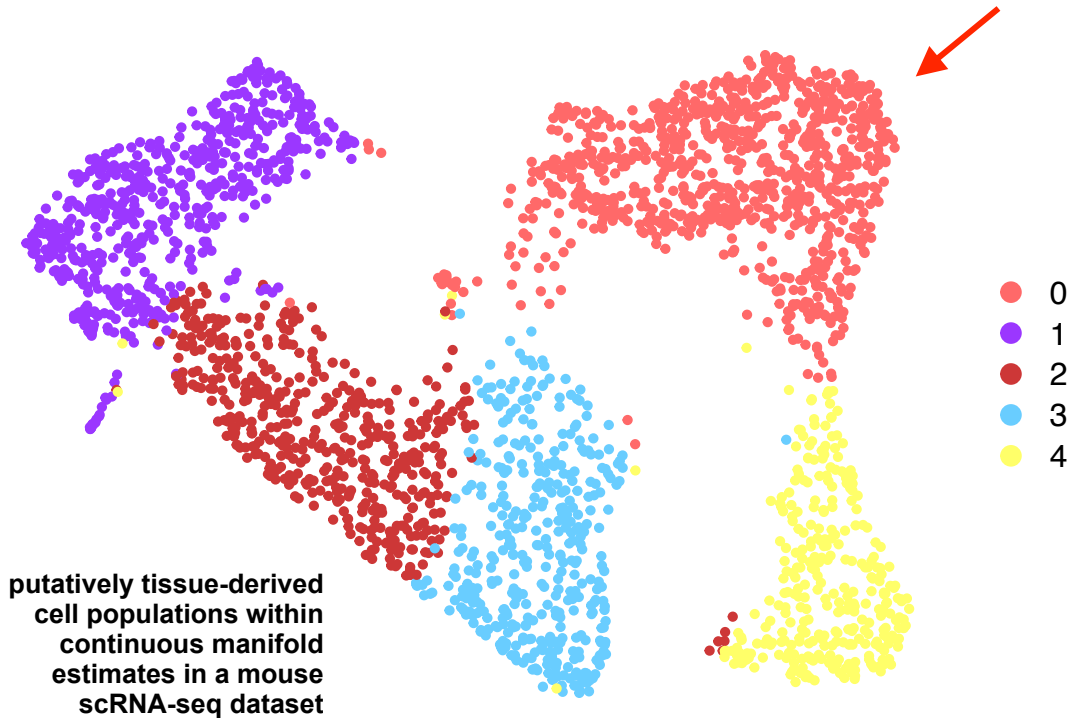

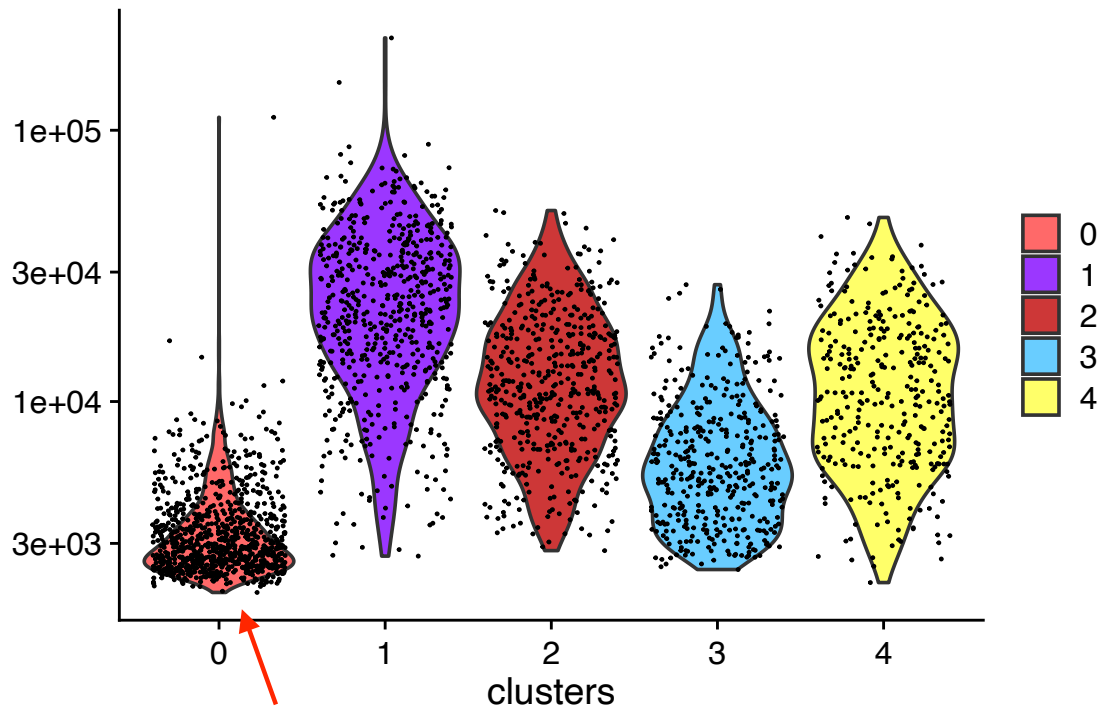

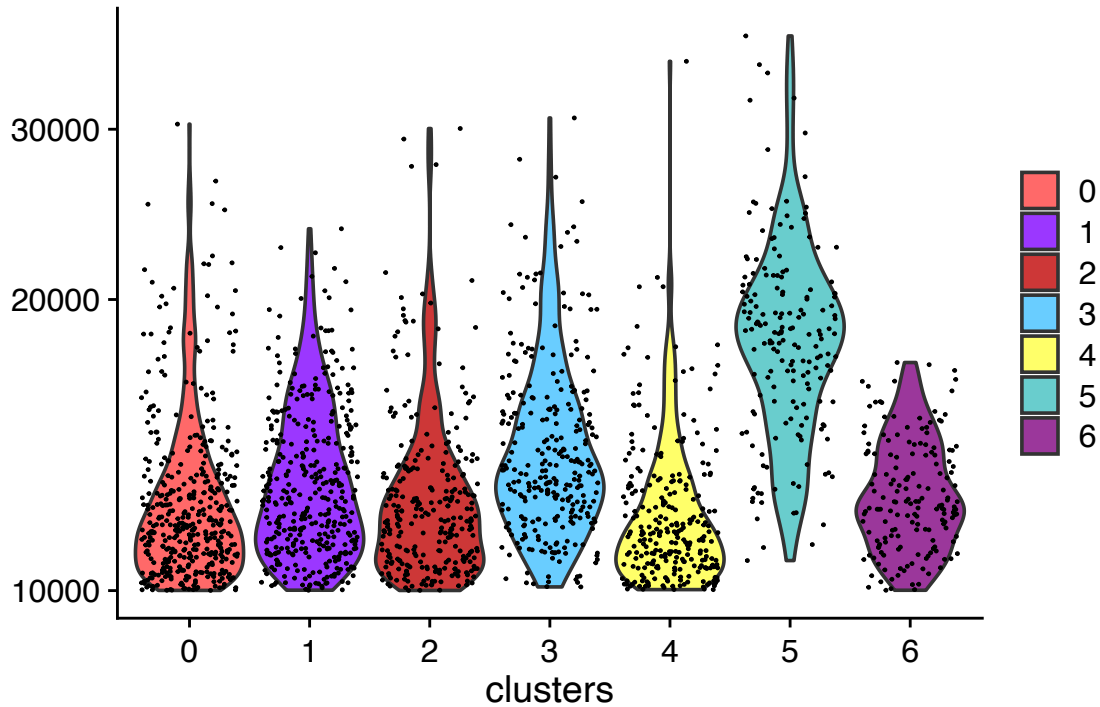

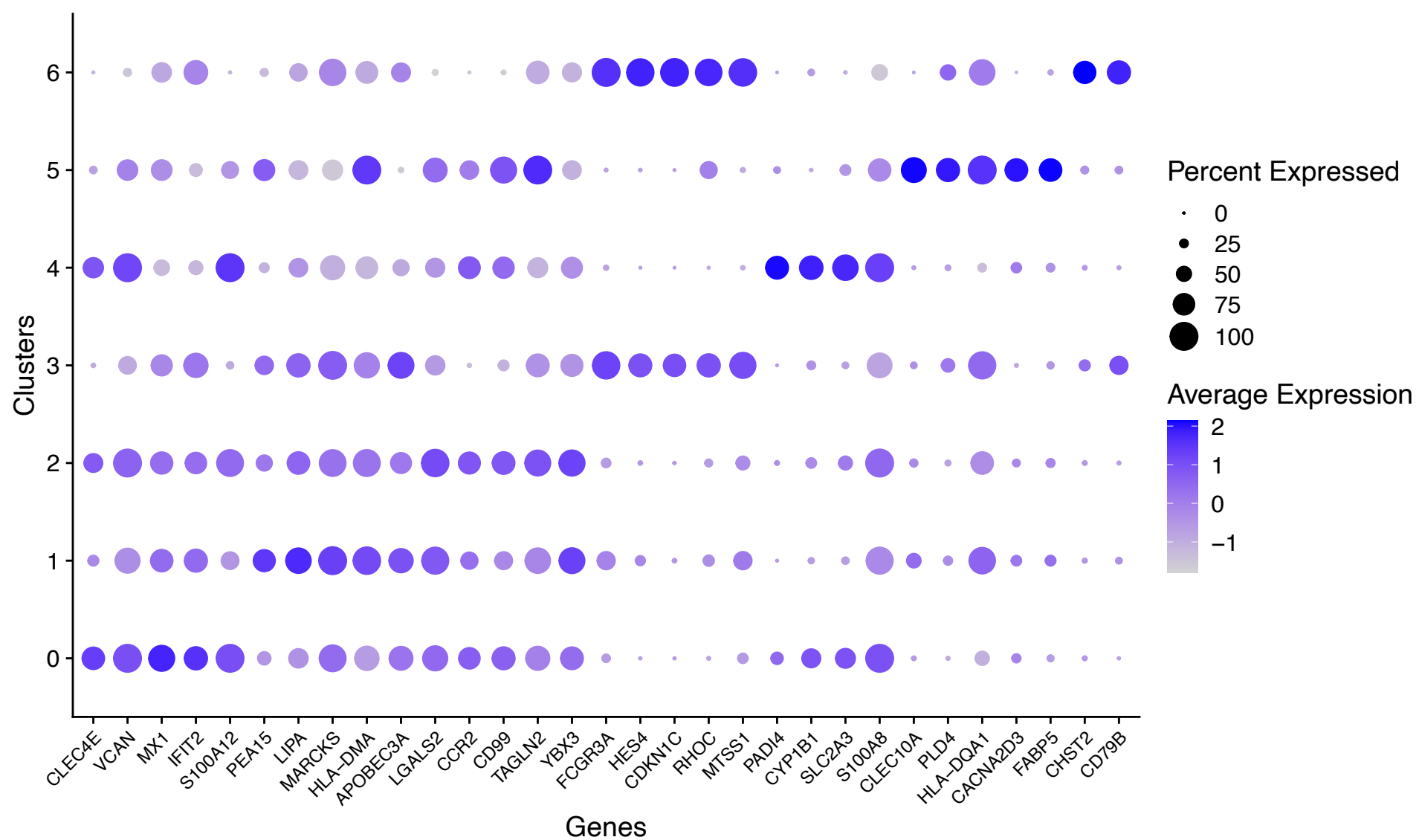

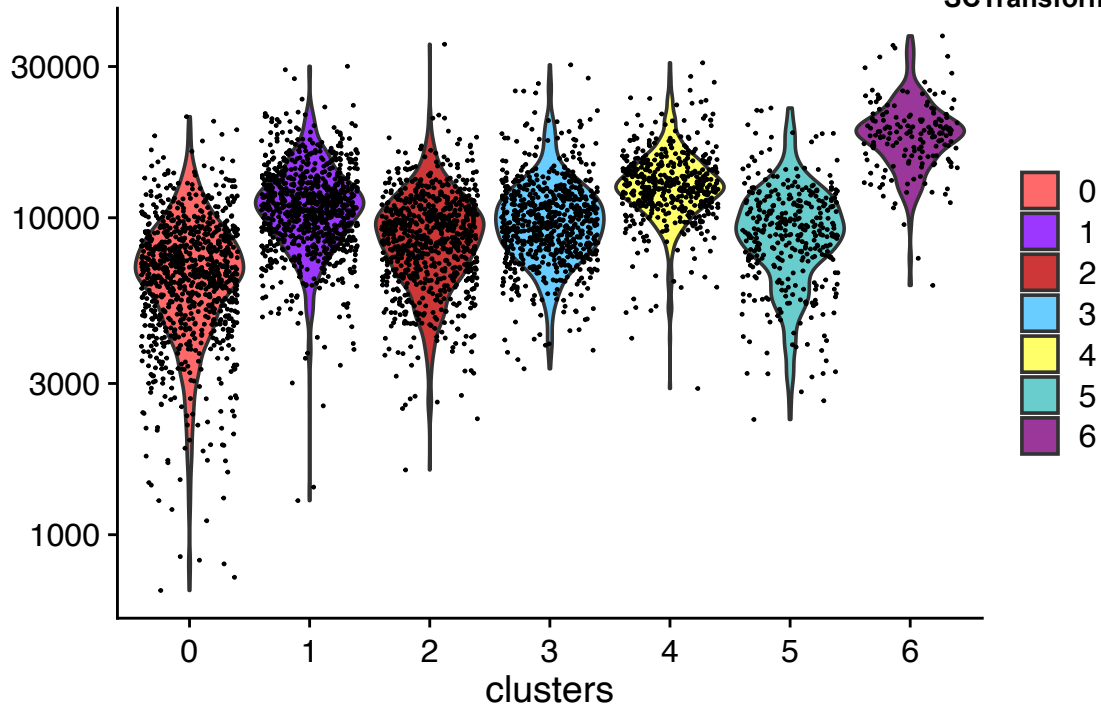

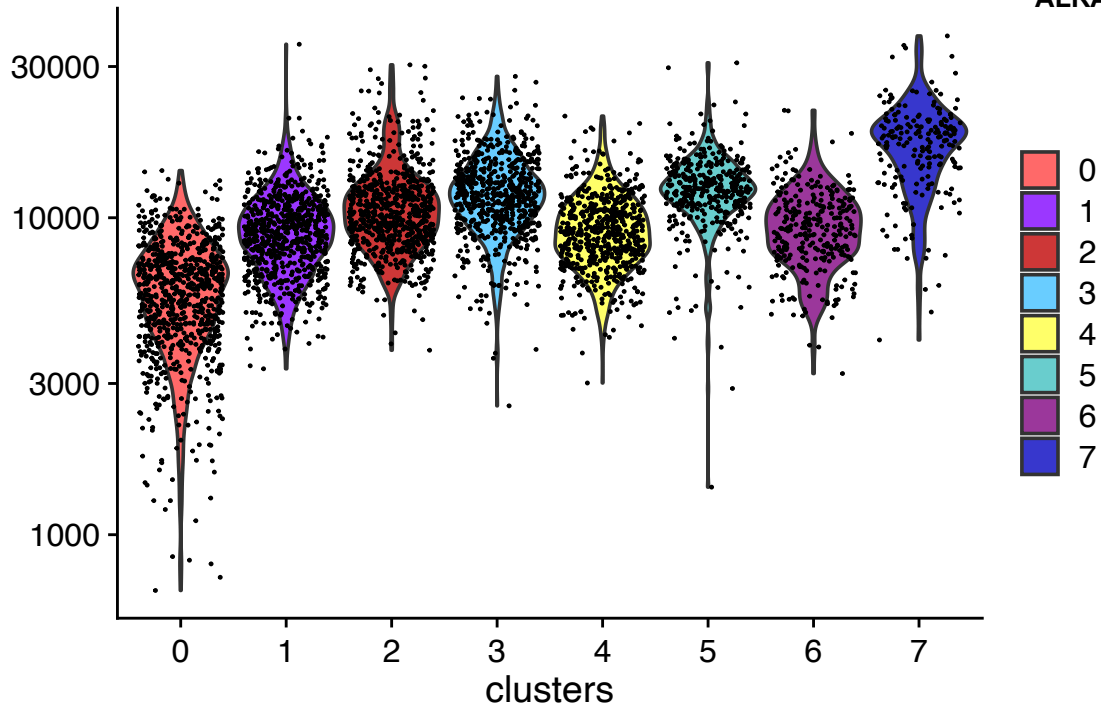

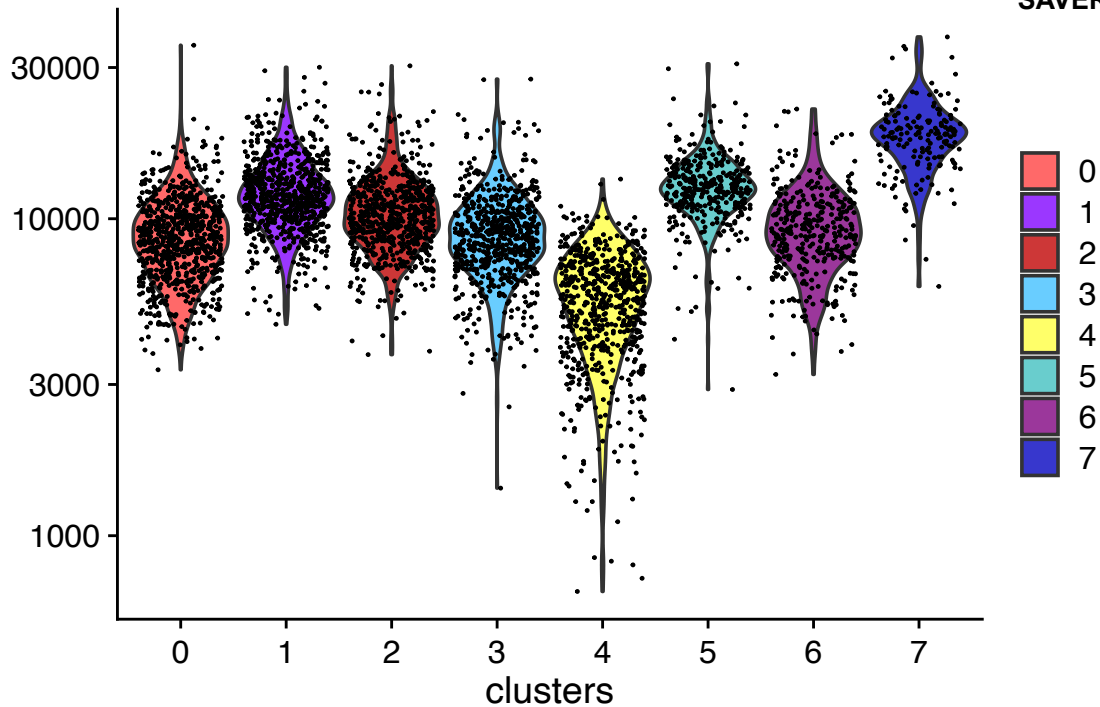

scImpute

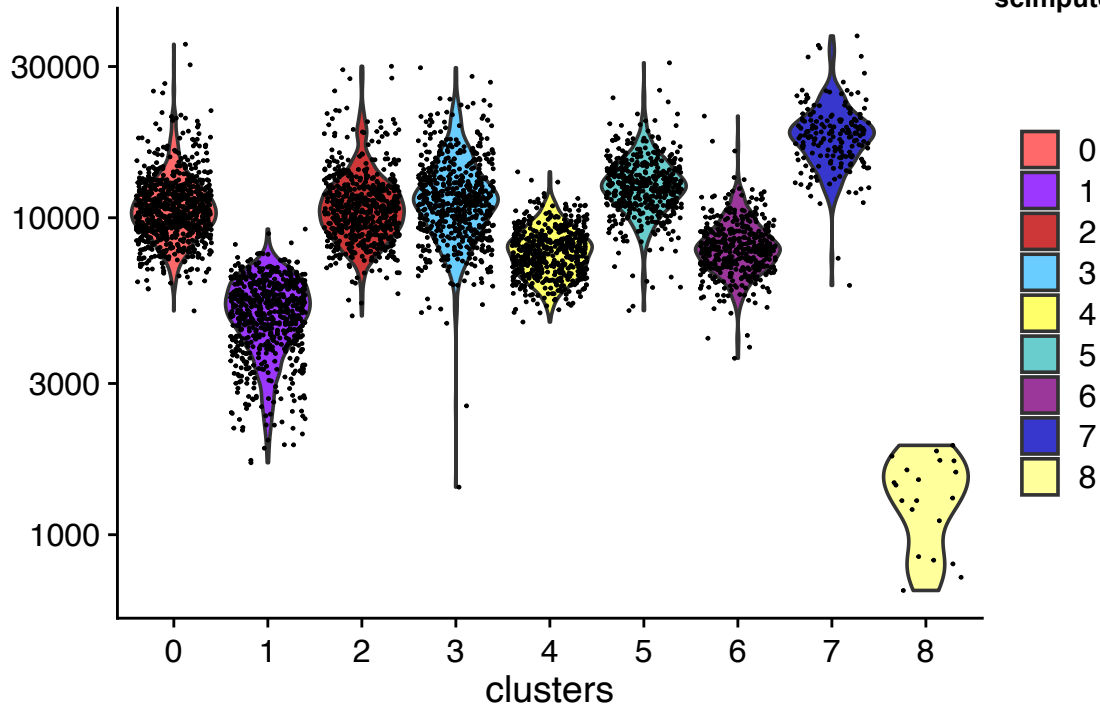

UMI Occurrence Frequency

0.000200  
0.000175  
0.000150  
0.000125  
0.000100  
0.000075  
0.000050  
0.000025  
0.000000

0

10000

20000

30000

Gene ID (i)

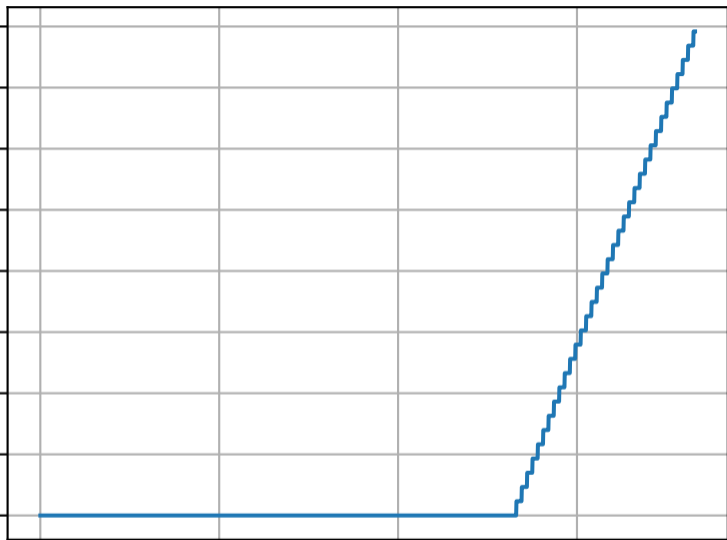

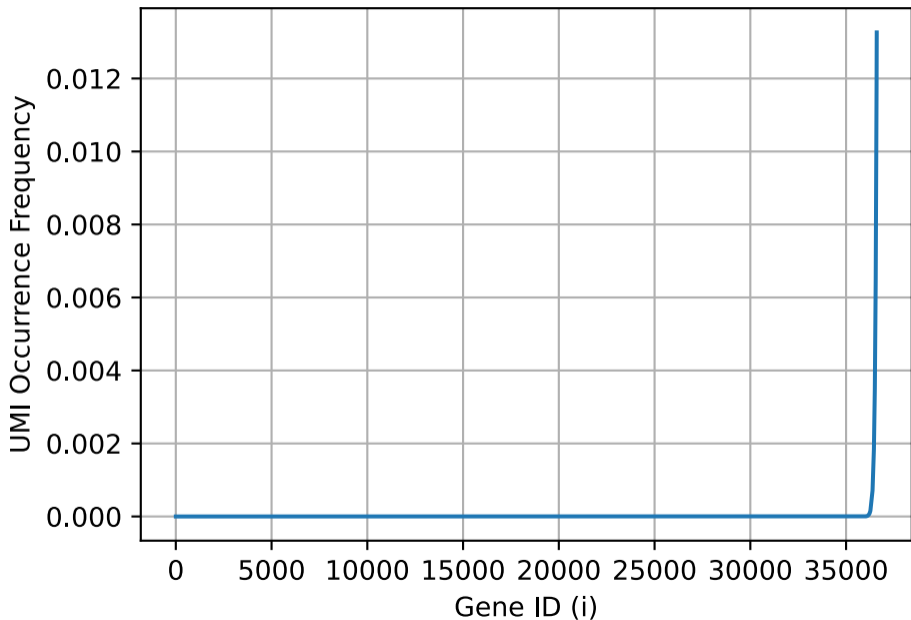

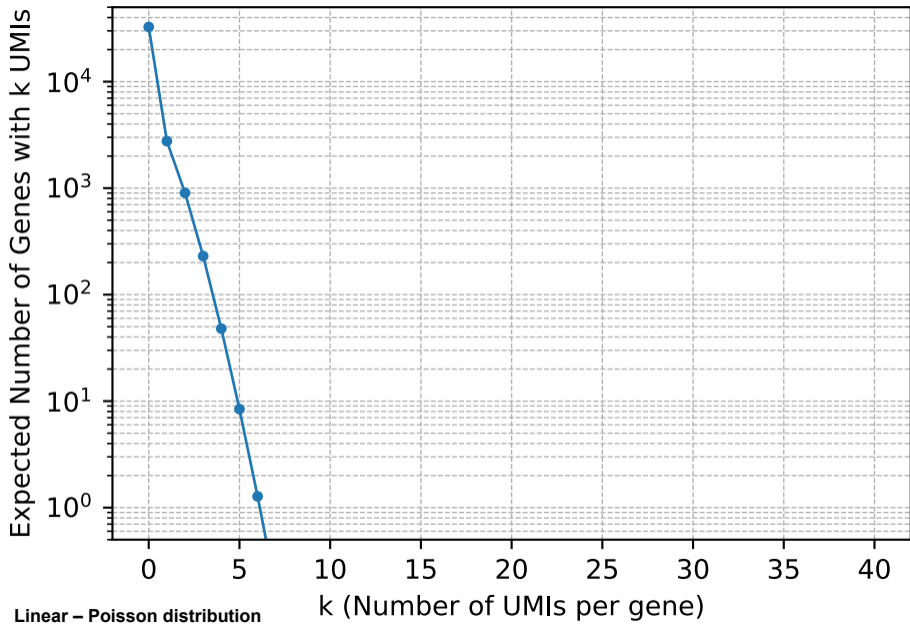

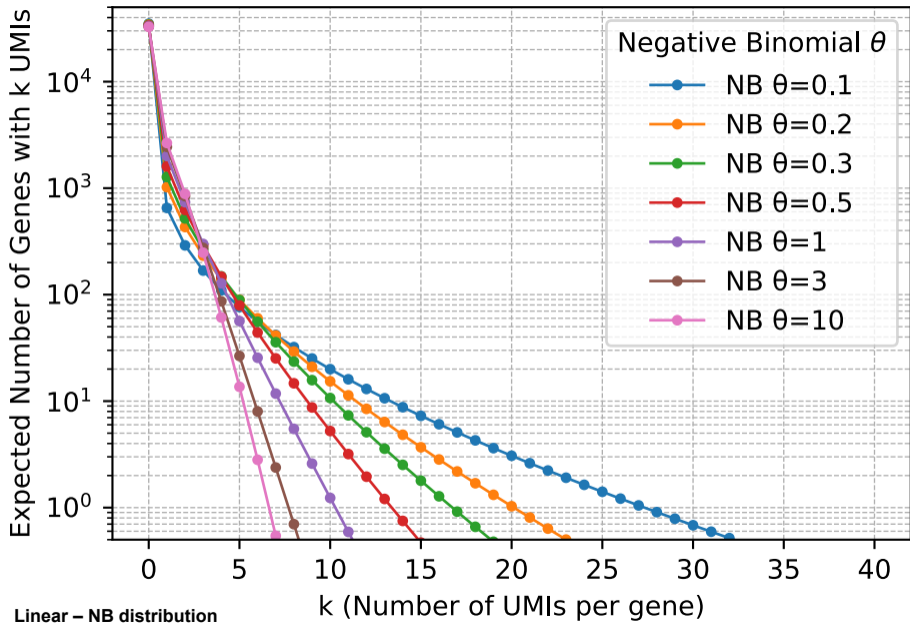

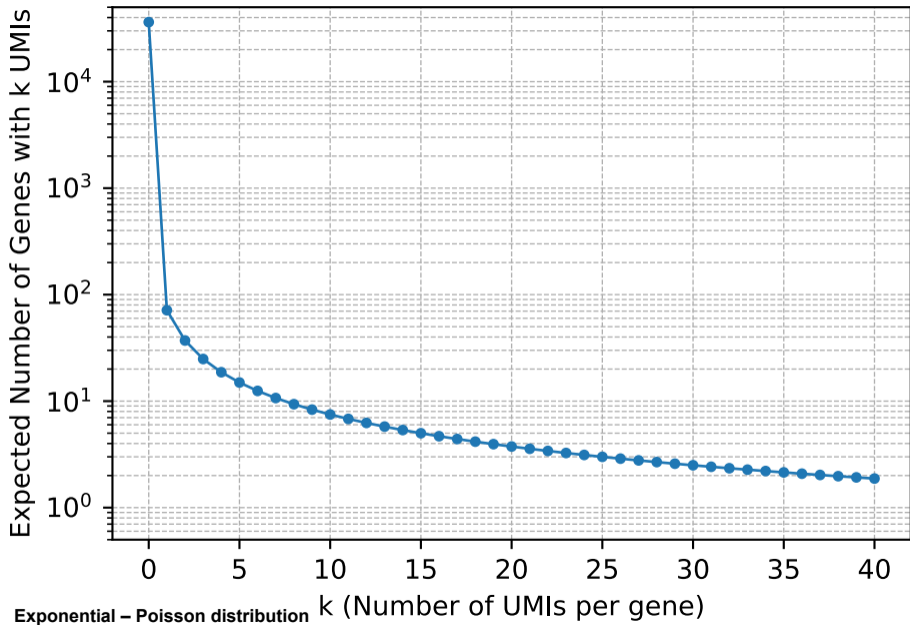

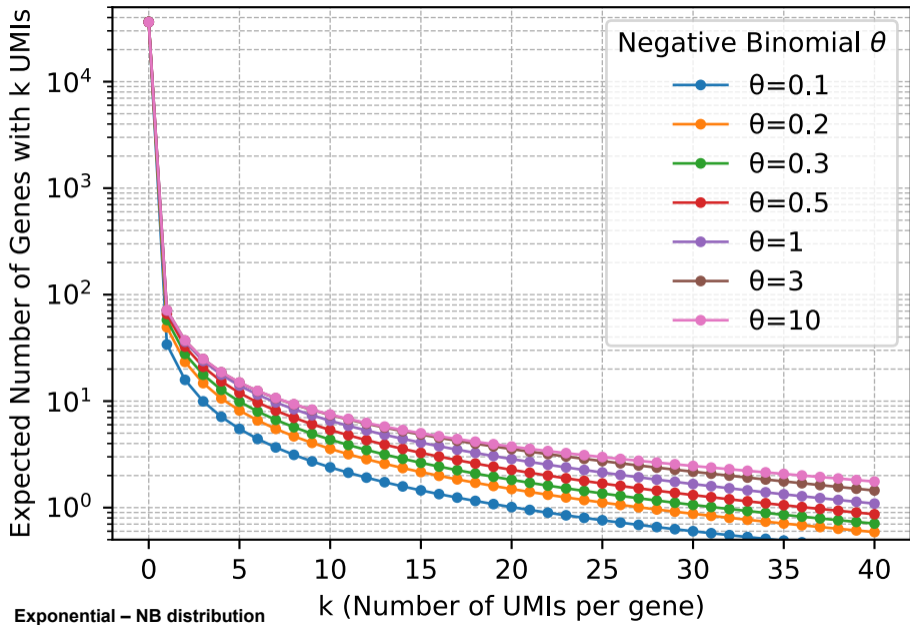

Supplement: Supplementary 1 — Figs. S1 to S4 [file csbj.0087.f1.zip › Integrated supplementary figures.pdf]
